# An Interesting Case

**Published:** 1894-01

**Authors:** L. Amster

**Affiliations:** Atlanta, Ga.


### AN INTERESTING CASE.

ATLANTA, GA., November, 1893.

*Editor Atlanta Medical and Surgical Journal:*

A case, or rather a series of cases, which seemed to me unique and of extraordinary occurrence, came under my observation last year in Macon, Ga.

Kindly allow me space in your journal for its report.

Mr. ———, aged thirty, married, came to my office to be treated for a case of gonorrhea, contracted during the absence of his wife on a visit North.

His wife having returned the same day I impressed upon him the urgent necessity of abstaining from coitus with her. Contrary to my admonition he had intercourse with her the very same night.

Several days after I was called to attend the wife, in consequence of her husband's criminal folly. She had a virulent case of gonorrhea. Both were cured in the course of about three weeks.

But this fatal intercourse, besides infecting the woman, had a "twin conception" as its results. At term I delivered her of a robust and well-developed boy, and twelve minutes after of a small and weakly girl. Knowing the history of the conception, I ordered antiseptic vaginal douches before delivery and dropped a solution of silver nitrate nitr. into the children's eyes, following delivery.

One week later the sickly girl infant developed a "gonorrheal ophthalmia," which responded to treatment and was entirely cured. Strange to say, none of the characters in this connubial drama were harmed by this triple gonorrheal infection. I ascertained a few days ago that the mother is healthy and both children alive and doing well. DR. L. AMSTER.

---

### PUERPERAL SEPSIS.

Dr. A. H. Cordier, Lecturer on Diseases of Women in the Kansas City Medical College, in discussing the question of puerperal sepsis in an article in the *Journal of the American Medical Association*, says: Many cases of puerperal sepsis develop regardless of any aseptic precautions on the part of the accoucheur. In the majority of cases in this type of the disease the foci of infection will be found in the Fallopian tubes. Fallopian tubes may be infected with the gonococci or other specific or pathogenic bacteria after impregnation. Pathogenic micro-organisms develop more rapidly and are more virulent during the puerperal period. This is due to the fact that (either) the secretions are more favorable culture fluids, or there exists a weakening of the resisting powers (want of immunity) on the part of the lying-in-woman. The obstetrician should rely upon the physical examination of the case under investigation in classifying the form of the disease he has to deal with. After deciding as to the location of the poisonous foci, the treatment at once suggests itself. If in doubt as to the source, he should begin with the milder procedure and watch closely the results of treatment. If permanent improvement does not soon take place, he should then direct his course in another direction. Surgery in these cases must be done *early*, *thoroughly*, and quickly, to insure the lowest rate of mortality. Delayed and imperfect work will, in these cases, always bring disastrous results.

